# Alternative Sweeteners Modify the Urinary Excretion of Flavanones Metabolites Ingested through a New Maqui-Berry Beverage

**DOI:** 10.3390/foods9010041

**Published:** 2020-01-03

**Authors:** Vicente Agulló, Raúl Domínguez-Perles, Diego A. Moreno, Pilar Zafrilla, Cristina García-Viguera

**Affiliations:** 1 Group of Quality, Safety and Bioactivity of Plant Foods, Department of Food Science and Technology, Phytochemistry and Healthy Foods Lab, (CEBAS-CSIC), University Campus of Espinardo 25, 30100 Murcia, Spain; vagullo@cebas.csic.es (V.A.); dmoreno@cebas.csic.es (D.A.M.); cgviguera@cebas.csic.es (C.G.-V.); 2 Department of Pharmacy, Faculty of Health Sciences, Universidad Católica San Antonio de Murcia (UCAM), Campus de los Jerónimos, 30107 Guadalupe, Murcia, Spain; mpzafrilla@ucam.edu

**Keywords:** dietary intervention, maqui-citrus juice, flavanones, urinary excretion, UHPLC-ESI-QqQ-MS/MS

## Abstract

Dietary sugar has been largely related to the onset of metabolic diseases such as type 2 diabetes and obesity, among others. The growing awareness on the close relationship between the dietary habits and this health disturbance has encouraged the development of new beverages using alternative sweeteners that could contribute to combat the above referred pathophysiological disorders. To gain further insight into this issue, the present work, upon an acute dietary intervention, evaluated the urinary excretion of flavanones ingested through polyphenols-rich beverages composed of maqui berry and citrus, with the aim of establishing the highest urinary excretion rate and metabolite profiles. The functional beverages evaluated were supplemented with a range of sweeteners including sucrose (natural and high caloric), stevia (natural and non-caloric), and sucralose (artificial and non-caloric) as an approach that would allow reducing the intake of sugars and provide bioactive phenolics (flavanones). The juices developed were ingested by volunteers (*n* = 20) and the resulting flavanones and their phase II metabolites in urine were analyzed by Ultra-High Performance Liquid Chromatography ElectroSpray Ionization Mass Spectrometry (UHPLC-ESI-MS/MS). A total of 16 metabolites were detected: eriodyctiol, naringenin, and homoeriodyctiol derivatives, where peak concentrations were attained 3.5 h after beverage intake. Sucralose and stevia were the sweeteners that provided the highest urinary excretion for most compounds. Sucrose did not provide a remarkable higher elimination through urine of any compounds in comparison with sucralose or stevia. These results propose two alternative sweeteners to sucrose (sucralose and stevia), an overused, high caloric sweetener that promotes some metabolic diseases.

## 1. Introduction

Recently, scientific evidence has shown that sugar intake stimulates brain reward pathways [1,2,3,4], the basis for the consumers choosing the types of foods and beverages that use sweeteners such as sucrose, glucose, and saccharine liquid, among others. This consumption pattern has been related with the rising incidence of metabolic disorders, such as type 2 diabetes, by promoting obesity and insulin resistance [5,6,7]. Associated to these pathophysiological processes, an increase of strokes has also been reported [8]. Thus, in this aspect, sweetened beverages constitute the main dietary source of sugars in the human diet [9].

Global strategies against the impact on health of sugar consumption are focused on establishing balanced diets that avoid the excessive consumption of sweeteners [10]. In connection with this strategy, a number of studies have suggested the consumption of bioactive molecules (phytochemical compounds, such as flavanones) which have a vasodilator activity that contributes with the improvement of endothelial function, and thus, can help prevent the deleterious effects associated with sugar [10]. However, the bioavailability of these bioactive compounds is generally low (up to 10% of the total intake) and could be strongly conditioned by the physico-chemical properties of the matrix (foods and beverages). Therefore, a recent study has proposed the use of alternative sweeteners such as stevia, as a trans-glycosylated food additive that could potentially improve the stability, bioaccessibility, and bioavailability of polyphenols [11]. In addition, the strategy of using other sweeteners is in agreement with the recommendations by the World Health Organization (WHO) to reduce the intake of free sugars to values less than 10% in order to diminish the array of pathophysiological disorders associated with their consumption [12].

To contribute to the enhanced consumption of dietary sources of bioactive phytochemicals, in the last few years diverse fruits have been selected and characterized for the development of new bioactive beverages, which have a high bioavailability and content of bioactive compounds, and contribute with lowering the risk of a range of diseases [13,14,15]. In the work developed by Gironés-Vilaplana et al. [13], Maqui (*Aristotelia chilensis* (Mol.) Stuntz), a purple blackberry from Chile and Argentina, was chosen due to its importance as a “superfruit” according to its health properties, such as a high antioxidant capacity, cardioprotection activities, and inhibition of adipogenesis, and diabetes symptoms [13].

In addition to red fruits, citrus fruits have been selected as ingredients of the new beverage due to their phenolic composition, as they are flavonoid-rich fruits. Citrus juices have significant effects for decreasing diastolic blood pressure, enhancing endothelium-dependent microvascular reactivity and increasing the pro-coagulant activity [16,17]. On the other hand, this type of beverage contains many bioactive nutrients and non-nutrients, which can provide health benefits beyond nutrition and cardiovascular disease, cancer, diabetes, and obesity [18]. The most abundant polyphenols in citrus are flavanones, with naringenin (N), eriodyctiol (E), and hesperidin (H) being the most representative compounds of this family, with the latter being promoted as a preventive molecule against cardiovascular diseases [16].

This article deals with the influence of diverse sweeteners on the urinary excretion of flavanones on healthy humans after an acute administration of a polyphenols-rich beverage composed of maqui berry and citrus, and created using three different sweeteners, including sucrose (natural and high caloric), stevia (natural and non-caloric), and sucralose (artificial and non-caloric). These sweeteners were selected to compare a classical, natural, and high-caloric sweetener and two non-caloric alternatives. Stevia was selected as a natural and emergent sweetener and sucralose as an artificial and widely used sweetener.

## 2. Material and Methods

### 2.1. Chemicals and Reagents

The standards used for quantification purposes, eriodyctiol (E), homoeriodictyol (HE), naringenin (N), and hesperidin (H) were purchased from TransMIT (Geiben, Germany). Formic acid and acetonitrile were obtained from Fisher-Scientific (Loughborough, UK). All solutions were prepared with ultrapure water from a Milli-Q Advantage A10 ultrapure water purification system (Millipore, Burlington, MA, USA).

### 2.2. Juice Preparation and Characterization of the Phenolic Content

Fresh dry organic maqui powder was provided by Maqui New Life S.A. (Santiago, Chile). Cítricos de Murcia S.L. (Ceutí, Spain) and AMC Grupo Alimentación Fresco y Zumos S.A. (Espinardo, Spain) provided the citrus juices. Sucrose was provided by AB Azucarera Iberia S.L. (Madrid, Spain), Stevia by AgriStevia S.L. (Murcia, Spain), and Sucralose by Zukan (Murcia, Spain).

For the manufacturing of maqui-citrus juices, maqui powder was mixed with citrus juices to obtain the base beverage. Then, the three selected sweeteners were added to obtain the different beverages characterized in the present work. The beverages underwent a pasteurization treatment by heating them at 85 °C for 58 s. Afterwards, the mixtures were bottled and stored at 5 °C until consumption by the volunteers.

The polyphenolic composition of the beverages was also characterized. With this objective, the juices were centrifuged at 10500 rpm for 5 min (Sigma 1–13, B. Braun Biotech International, Osterode, Germany). The supernatants were filtered through a 0.45 mm polyvinylidene fluoride (PVDF) filter (Millex HV13, Millipore, Bedford, MA, USA) and analyzed by RP-HPLC-DAD. The identification and quantification of flavanones was performed by applying a previously-used method [13,19]. Briefly, chromatographic analyses of the samples for the identification and quantification of flavanones were carried out in a Luna 5 µm C18(2)100 Å column (250 mm × 4.6 mm), using Security Guard Cartridges PFD 4 mm × 3.0 mm both supplied by Phenomenex (Torrence, CA, USA). The solvents used for the chromatographic separation were Milli-Q water/formic acid (95.0:5.0, *v*/*v*) (solvent A) and methanol (solvent B), with a linear gradient (time (min.), %B) (0, 15%); (20, 30%); (30, 40%); (35, 60%); (40, 90%); (44, 90%); (45, 15%); and (50, 15%), using an Agilent Technologies 1220 Infinity Liquid Chromatograph, equipped with an auto-injector (G1313, Agilent Technologies) and a Diode Array Detector (1260, Agilent Technologies, Santa Clara, CA, USA). Chromatograms were recorded and processed on an Agilent ChemStation for LC 3D systems. The volume of injection and flow rate were 10 µL and 0.9 mL/min, respectively. The quantification of flavanones was done on UV chromatograms recorded as hesperidin at 280 nm and expressed as mg per 100 mL of juice.

### 2.3. Experimental Design

A double-blind, randomized, cross-sectional clinical study was conducted on overweight people (*n* = 20). The study and protocol were approved by the official Ethical Committee of Clinical Studies (CEIC) of the General University Hospital Morales Meseguer (Murcia), and registered at ClinicalTrials.gov (NCT04016337). The volunteers provided written consent to participate in this study. The criteria for the volunteers’ selection for the study were to be in good health, overweight (between 24.9 and 29.9 kg/m^2^ following WHO criteria), aged 40–60 years, non-smokers, non-dyslipidemic and normotense, with no chronic illnesses and not taking any medication. After an initial wash-out phase of 3 days with a strict diet free of polyphenols and added sugars, 330 mL of the test drinks (stevia, sucralose, and sucrose as the added sweetener) were administered on fasting conditions. Urine samples were collected 24 h prior (0 point), as well as in the following intervals: 0–3.5 h, 3.5–12.0 h, and 12.0–24.0 h. After 15 days, the process was repeated again, with the volunteers ingesting another drink developed with the remaining sweetener, until all the drinks were consumed by all the volunteers (3 rounds). The urine samples collected were stored at −80 °C until analysis. The analysis was performed once each period was finished and in the same batch to minimize analytical variations.

The total volume of each urine interval was recorded to calculate the absolute amounts of the compounds and metabolites excreted in the study period. Also, creatinine content was determined to normalize the concentrations of metabolites in urine as µg compound/mg creatinine, to control for differences in urine volumes.

### 2.4. Urine Samples Collection, Processing, and Analysis by UHPLC-ESI-MS/MS

Urine samples were defrosted and diluted 1:2 (*v*/*v*) in MilliQ-water/formic acid (99.9:0.1, *v*/*v*) and centrifuged at 15,000× *g* for 10 min, at 5 °C (Sigma 1–16, B. Braun Biotech International, Osterode, Germany). Afterwards, supernatants were filtered through 0.45 µm PVDF filters (Millex HV13, Millipore, Bedford, MA, USA) and stored at −20 °C until analysis by Ultra-High Performance Liquid Chromatography ElectroSpray Ionization Mass Spectrometry (UHPLC-ESI-MS/MS).

The identification and quantification of flavanone metabolites was performed by applying the method previously reported by Medina et al. with some modifications [20]. The analysis of samples on the profile and concentration of anthocyanin metabolites was carried out on with an Ascentis Express F5 column (5 cm × 2.1 mm; 2.7 µm) (Sigma, Osterode, Germany). The solvents used for the chromatographic separation were Milli-Q water/formic acid (99.9:0.1, *v*/*v*) (solvent A) and acetonitrile/formic acid (99.9:0.1, *v*/*v*) (solvent B), with a linear gradient (time (min.), %B) (0, 10%); (1, 10%); (10, 60%); (11, 80%); (13, 80%); (13.01, 10%), and (14.50, 10%); using an UHPLC system coupled with a triple quadrupole tandem mass spectrometer model 6460 (Agilent Technologies, Waldbronn, Germany), operating in multiple reaction monitoring (MRM) and negative/positive electrospray ionization (ESI) modes. The volume injected and flow rate were 10 µL and 0.2 mL/min, respectively. The MS parameters, at the optimized conditions, were gas temperature 325 °C; gas flow 10 L/min; nebulizer 40 psi; sheath gas heater 275 °C; sheath gas flow 12; capillary voltage 4000–5000 V; Vcharging 1000–2000. Data acquisition and processing were performed by using MassHunter software version B.08.00 (Agilent Technologies, Walbronn, Germany).

### 2.5. Statistical Analysis

Quantitative data are presented as mean ± SD of 20 volunteers. Specific differences were examined by an analysis of variance (ANOVA) and a multiple range test (Duncan’s test). The data were processed using the SPSS 21.0 software package (SPSS Inc., Chicago, IL, USA.) and the level of significance was set at *p* < 0.05.

## 3. Results and Discussion

### 3.1. Flavanone Content of Juices

In order to establish the rate of urinary elimination of flavanones present in the maqui-citrus juices manufactured using three separate sweeteners (stevia, sucralose, and sucrose), their flavanones profile and concentration in juices were measured. In this regard, the presence of four flavanones was observed, which were found in the following decreasing concentration in all juices, H-rutinoside (4.87 mg/100 mL, on average) > N-rutinoside (1.31 mg/100 mL, on average) > E-rutinoside (0.32 mg/100 mL, on average) > N-hexoside derivatives (0.14 mg/100 mL, on average). As shown in Table 1, no significant differences were observed between the flavanone content of the maqui-citrus juices developed using diverse sweeteners either when considering individual or total flavanones.

### 3.2. Qualitative Analysis of Urine Metabolites of Flavanones from Maqui-Citrus Juice

To profile the metabolites of flavanones excreted in urine, 24-h urine collected after the ingestion of 330 mL of maqui-citrus juice by healthy volunteers were processed, allowing for the evaluation of the differences due to the sweetener employed in the development of the juices (stevia, sucralose, and sucrose). The analysis results show that the urine samples exhibited 16 diverse phenolic metabolites, derived from the list of flavanones present in the juices and shown in Table 2. More specifically, the compounds identified in the urine samples were E, E-glucuronide, E-sulfate, E-disulfate, HE, HE-glucuronide, HE-diglucuronide, HE-sulfate, HE-glucuronide-sulfate, N, N-glucoside, narirutin, N-glucuronide, N-diglucuronide, N-sulfate, and N-glucuronide-sulfate. Interestingly, neither H, nor its phase II derivative, were detected, although H was the main flavanone in the maqui-citrus based beverages, accounting for 73.3% of the total flavanones, on average.

Since the metabolism of the precursors of flavanone metabolites is closely dependent on the metabolic traits of the volunteers and the inter-individual variation [21], a range of metabolites (E, E-disulfate, HE, HE-diglucuronide, and HE-glucuronide-sulfate) was found in urine of a reduced number of volunteers. These compounds were present in quantifiable amount but the limited number of volunteers excreting these molecules indicates that these were not representative. The inter-individual variation could also be responsible for the dispersion of the concentration of the metabolites, making difficult the identification of significant differences relative to the quantitative determinations. On the contrary, E-glucuronide, E-sulfate, HE-glucuronide, HE-sulfate, N, N-glucoside, narirutin, N-glucuronide, N-diglucuronide, N-sulfate, and N-glucuronide-sulfate were identified and quantified in the urine from all volunteers.

### 3.3. Quantification of Flavanone Metabolites in Urine Samples

**Figure 1:**
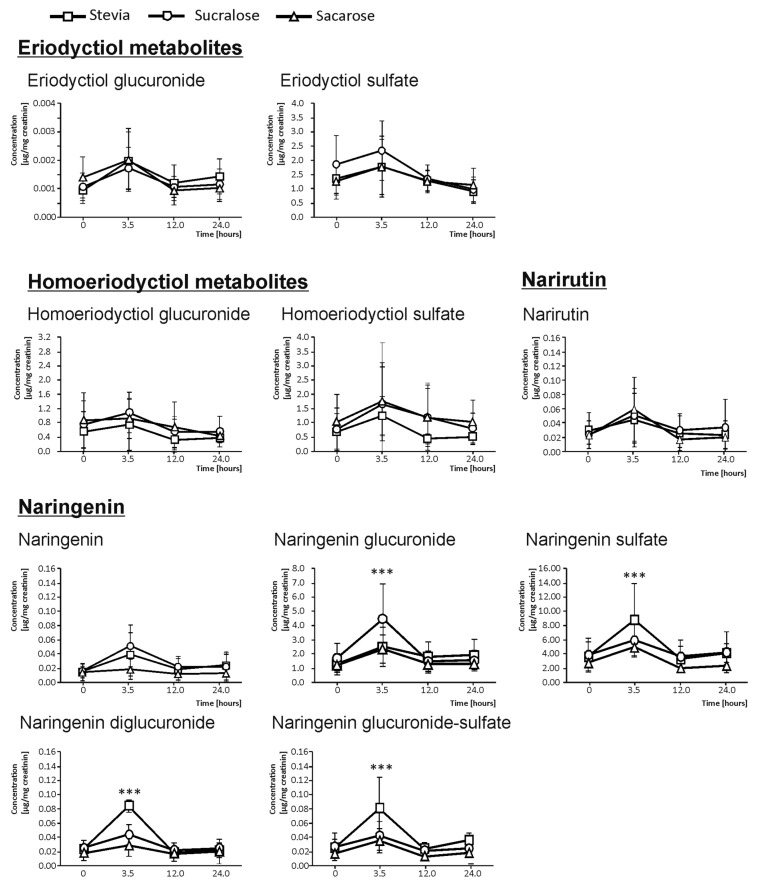
Content (mean ± SD, *n* = 2) of single flavanone metabolites in basal urine and 3.5, 12, and 24-h urine of healthy volunteers after ingesting 330 mL of maqui-citrus juices developed using as sweeteners stevia (□), sucralose (O), and sucrose (∆). Significantly different bioavailabilities according to an analysis of variance (ANOVA) and Duncan’s multiple rank test were found at *p* < 0.001 (***).

In urine, the quantification of the flavanone metabolites excreted was based on basal urine (0 h), as well as on urine excreted between 0 and 3.5 h, 3.5–12.0 h, and 12.0–24.0 h. The excretion kinetics for E and N matched, showing the highest concentration at 3.5 h after the intake of the beverages (Figure 1). For this reason, all the concentrations described below referred to 3.5 h after the ingestion. In the case of HE derivatives, no significant increases in the urine concentration was found.

The sum of E and their phase II conjugates excretion provided values of 0.18 µg/mg creatinine in volunteers ingesting the beverages developed using sucralose as the sweetener. This concentration was higher than the one reached when using stevia and sucrose. The beverages developed using the latter sweeteners showed a 9.9% lesser amount of excretion than sucralose, on average. However, despite the trend observed, the large variation between volunteers did not allow for providing significant differences [21].

As mentioned above, the other flavanone metabolites present in urine in quantifiable concentrations were the N derivatives (Figure 1). The sum of N and their phase II derivatives excretion values was 115.91 µg/mg creatinine for sucralose, resulting in 12.2% and 32.1% lower amounts than sucralose, respectively, of stevia and sucrose-sweetened juices. When analyzing the effect of the sweetener in regard to the individual compounds, for N-diglucuronide, N-glucuronide-sulfate, and N-sulfate, the highest value corresponded to juices developed using stevia as a sweetener, which gave rise to 1.45, 1.69, and 19.74 µg/mg creatinine concentrations, respectively. Indeed, these concentrations significantly surpassed the urinary excretion reached when juices sweetened with sucralose and sucrose were ingested, by 33.3% (N-diglucuronide), 38.9% (N-glucuronide-sulfate), and 25.3% (N-sulfate), on average. In respect to N-glucuronide, the highest value corresponded to sucralose (93.71 µg/mg creatinine), which significantly surpassed the urine concentration provided by stevia and sucrose (25.2% lower, on average).

Moreover, stevia was the sweetener that provided a higher urinary excretion for most compounds derived from N (N-diglucuronide, N-glucuronide-sulfate, and N-sulfate), followed by sucralose (N-glucuronide). Sucrose did not provide remarkable higher rates of elimination through urine of any compounds in comparison with sucralose or stevia, as intestinal sugar carriers may play an important role in flavonoid absorption [22].

The results suggested that both stevia and sucralose were better than sucrose in terms of urinary excretion. Several studies of the effects on human health and metabolic diseases of stevia and sucralose showed contradictory results, as extensively reviewed by Daher et al. This author indicated that most intervention studies have assessed the role of isolated non-nutritional sweeteners and not as part of a habitual diet [23]. Thus, further studies are needed to learn more about the influence of stevia and sucralose on human health.

## 4. Conclusions

The results of the present work evidence that processing in respect to the selection of sweeteners does not seem to have any effect on flavanone concentration. On the other hand, the absorption rate of flavanones from citrus, excluding H, as they pass through the digestion system, is achieved through the formation of a variety of phase II derivatives. The results obtained pointed out sucralose and stevia as the sweeteners that had the greatest urinary excretion of N and most of its metabolites, which constitutes, as far as demonstrated responsible for valuable biological activities, a very useful marker for establishing the actual biological and healthy potential of the juices developed. Actually, they significantly surpassed the urinary excretion provided by sucrose that could be related with a variety of impacts of the diverse sweeteners on the actual bioavailability of flavanones. However, sucrose did not provide a higher urinary excretion as compared to sucralose or stevia in any of the cases.

This information would allow designing further studies of dietary interventions aimed at evaluating such sweeteners, which have gained relevance for human health. Indeed, considering the differences on urinary excretion between sweeteners, this study proposes two non-caloric sweetener alternatives (sucralose and stevia) in order to reduce the consumption of sucrose, a high caloric sweetener with an evident influence on metabolic disorders (type 2 diabetes and obesity, among others). Nevertheless, more studies are needed in order to better understand the effects on health of the two alternatives.

## Figures and Tables

**Table 1:** Flavanones composition of the maqui-citrus juices developed using diverse sweeteners.

Beverages	Flavanones ^Z^ (mg/100 mL)				
	N-Hexoside Derivated	E-Rutinoside	N-Rutinoside	H-Rutinoside	Total
Stevia	0.15 ± 0.02	0.32 ± 0.04	1.30 ± 0.01	4.87 ± 0.01	6.64 ± 0.2
Sucralose	0.14 ± 0.02	0.32 ± 0.01	1.31 ± 0.01	4.86 ± 0.01	6.63 ± 0.1
Sucrose	0.14 ± 0.01	0.31 ± 0.03	1.31 ± 0.01	4.88 ± 0.01	6.64 ± 0.1
*p*-value	>0.05 ^N.s.^	>0.05 ^N.s.^	>0.05 ^N.s.^	>0.05 ^N.s.^	>0.05 ^N.s.^

^Z^ N, naringenin; E, eriodyctiol; H, hesperetin. N.s., sot significant.

**Table 2:** Qualitative analysis of flavanone metabolites in urine after the ingestion of maqui-citrus juices.

Compound	R.T. (min)	Precursor Ion	Product Ion	Fragmentation (V)	C.E. (V)	Polarity
*Eriodyctiol metabolites*						
Eriodyctiol (E)	6.49	287.0	151.0	70	10	Negative
Eriocitrin	N.f.	449.0	287.0	70	10	Negative
E-glucuronide	4.87	463.0	287.0	70	10	Negative
E-di-glucuronide	N.f.	639.0	287.0	70	10	Negative
E-sulfate	5.53	367.0	287.0	70	10	Negative
E-di-sulfate	4.24	447.0	287.0	70	10	Negative
E-glucuronide-sulfate	N.f.	543.0	287.0	70	10	Negative
*Hesperetine metabolites*						
Hesperetine (H)	7.30	302.0	151.0	70	20	Negative
Hesperidin	N.f.	609.0	302.0	70	20	Negative
H-glucuronide	N.f.	478.0	302.0	70	20	Negative
H-di-glucuronide	N.f.	664.0	302.0	70	20	Negative
H-sulfate	N.f.	382.0	302.0	70	20	Negative
H-di-sulfate	N.f.	462.0	302.0	70	20	Negative
H-glucuronide-sulfate	N.f.	558.0	302.0	70	20	Negative
*Homoeriodyctiol metabolites*						
Homoeriodyctiol (HE)	7.30	301.0	151.0	110	15	Negative
HE-glucuronide	5.50	477.0	301.0	110	15	Negative
HE-di-glucuronide	4.22	653.0	301.0	110	15	Negative
HE-sulfate	5.90	381.0	301.0	110	15	Negative
HE-di-sulfate	N.f.	461.0	301.0	110	15	Negative
HE-glucuronide-sulfate	4.67	557.0	301.0	110	15	Negative
Naringenin (N)	7.26	271.0	119.0	130	20	Negative
N-glucoside	4.63	433.0	271.0	130	20	Negative
Narirutin	4.86	579.0	271.0	130	20	Negative
N-glucuronide	5.07	433.0	271.0	130	20	Negative
N-di-glucuronide	4.09	623.0	271.0	130	20	Negative
N-sulfate	5.90	351.0	271.0	130	20	Negative
N-di-sulfate	N.f.	431.0	271.0	130	20	Negative
N-glucuronide-sulfate	4.87	527.0	271.0	130	20	Negative

C.E., collision Energy; N.f.—not found; R.T., retention time.
